# Supplementary material for: Transcriptomic analysis of seed development in Paysonia auriculata (Brassicaceae) identifies genes involved in hydroxy fatty acid biosynthesis
Source: Front Plant Sci. 2023 Jan 13;13:1079146. doi: 10.3389/fpls.2022.1079146 (PMC9880434; doi:10.3389/fpls.2022.1079146)

Supplementary File 2. GC chromatograms and mass spectra of HFAs and FAs

2.1 14 Days after pollination (3 biological replicates)

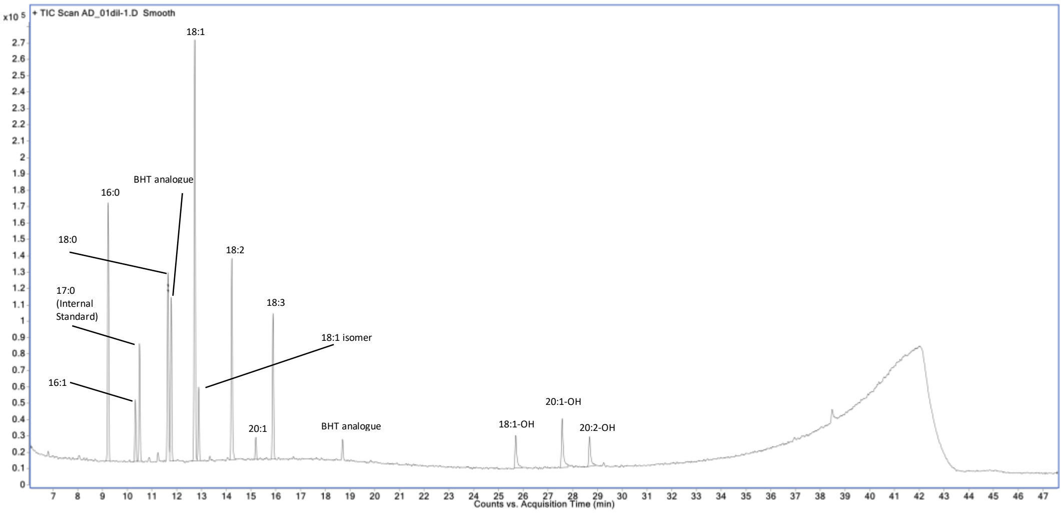

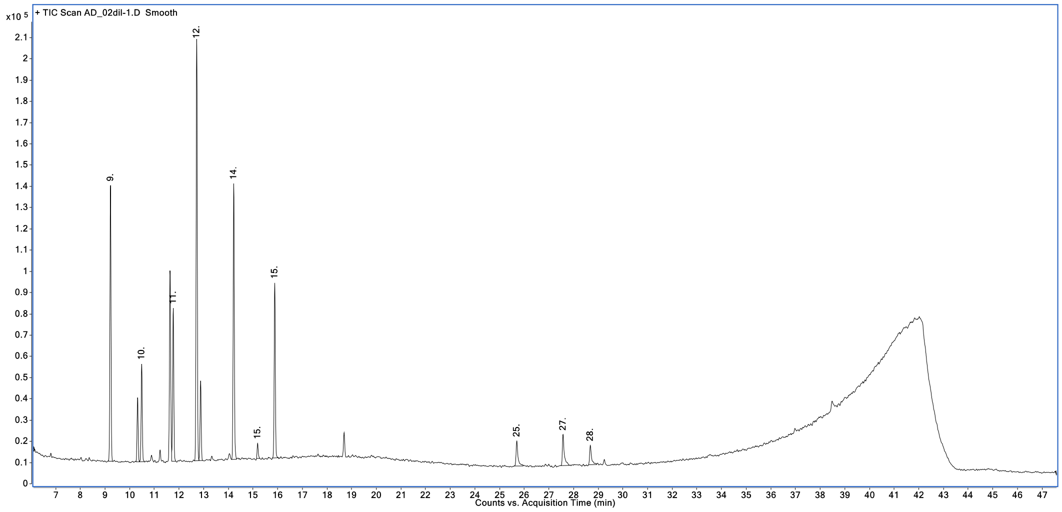

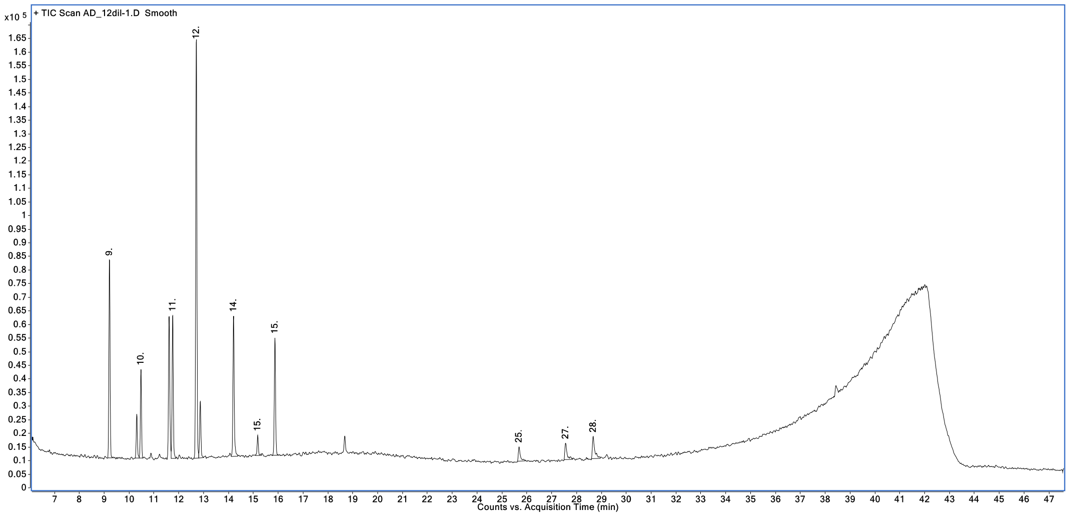

2.2 21 Days after pollination (3 biological replicates)

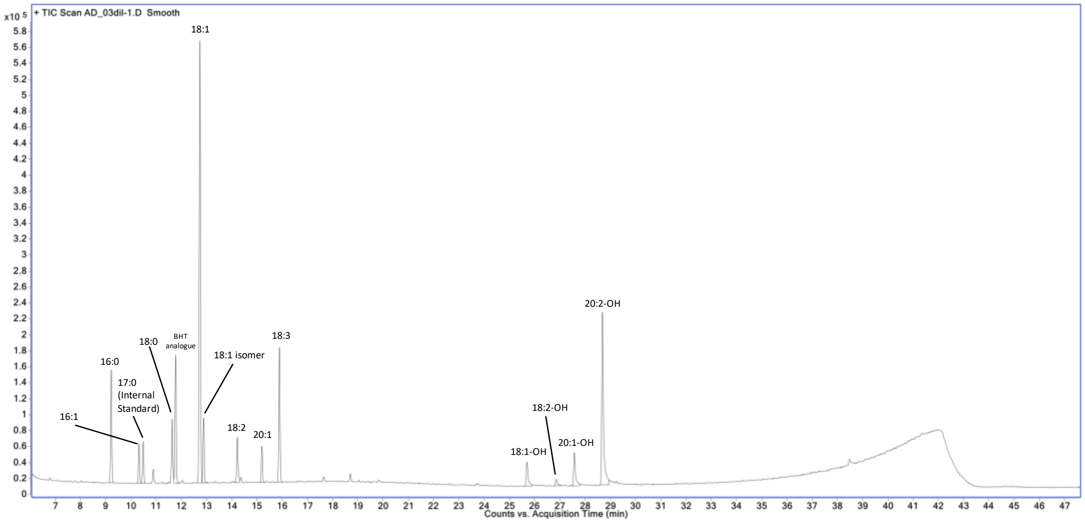

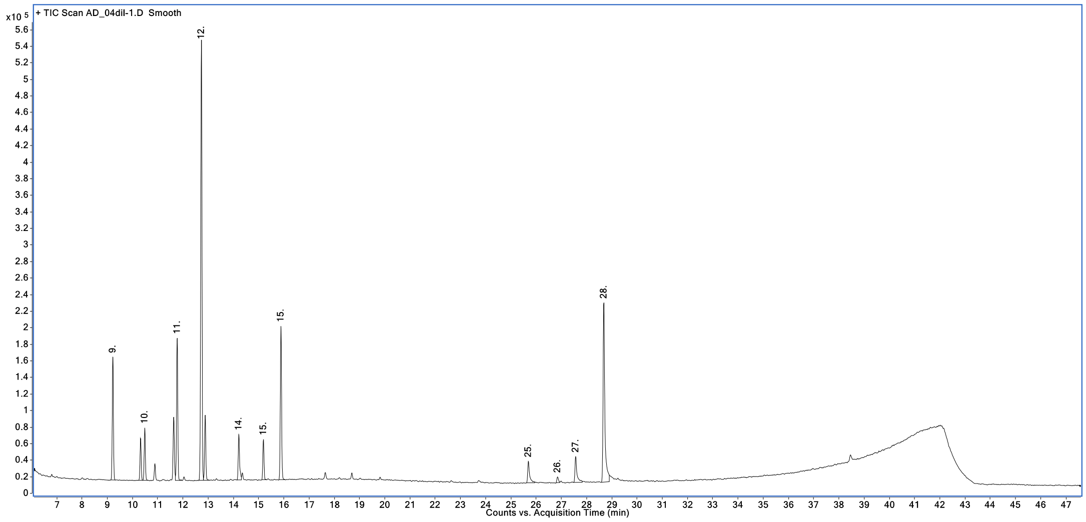

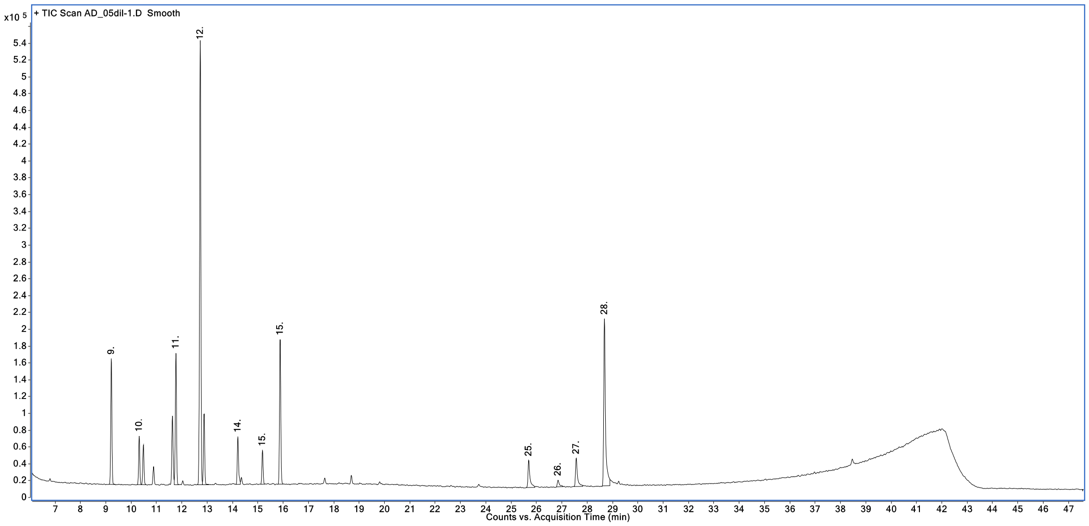

2.3 28 Days after pollination (3 biological replicates)

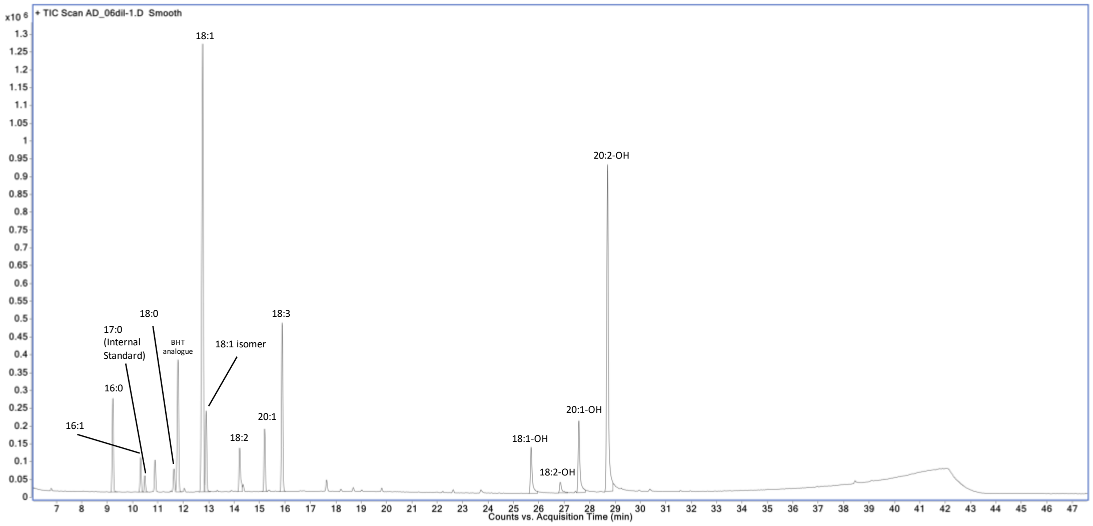

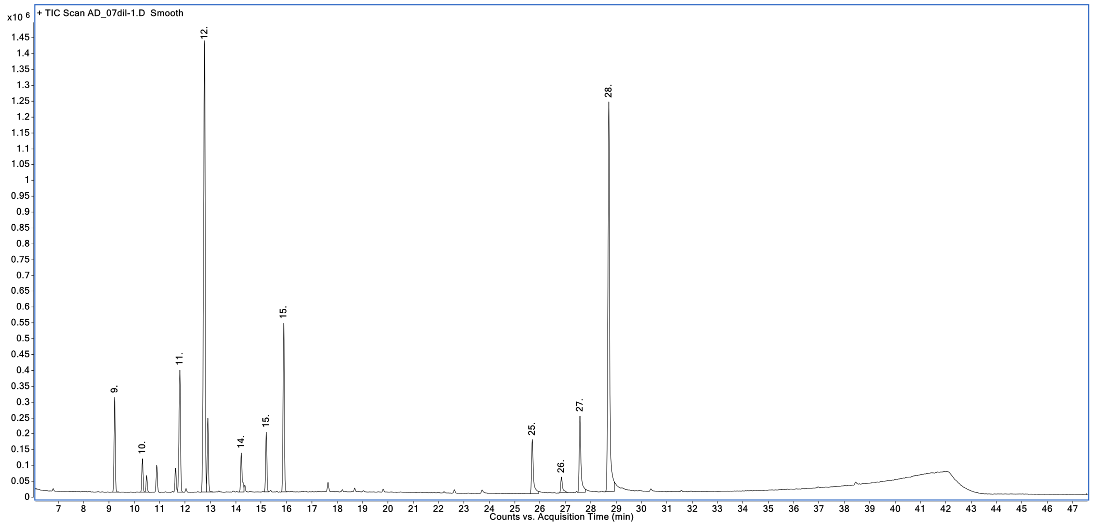

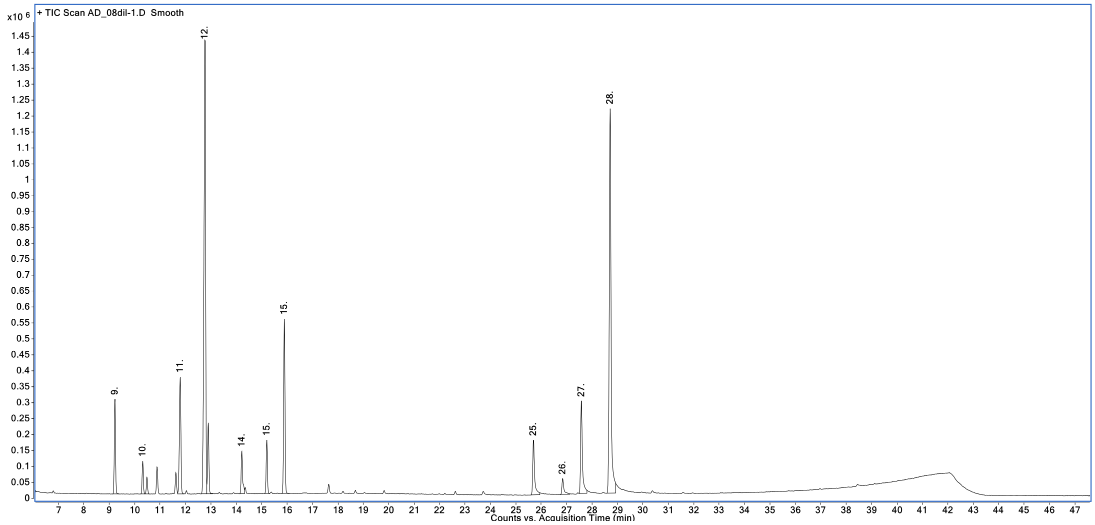

2.4 42 Days after pollination (3 biological replicates)

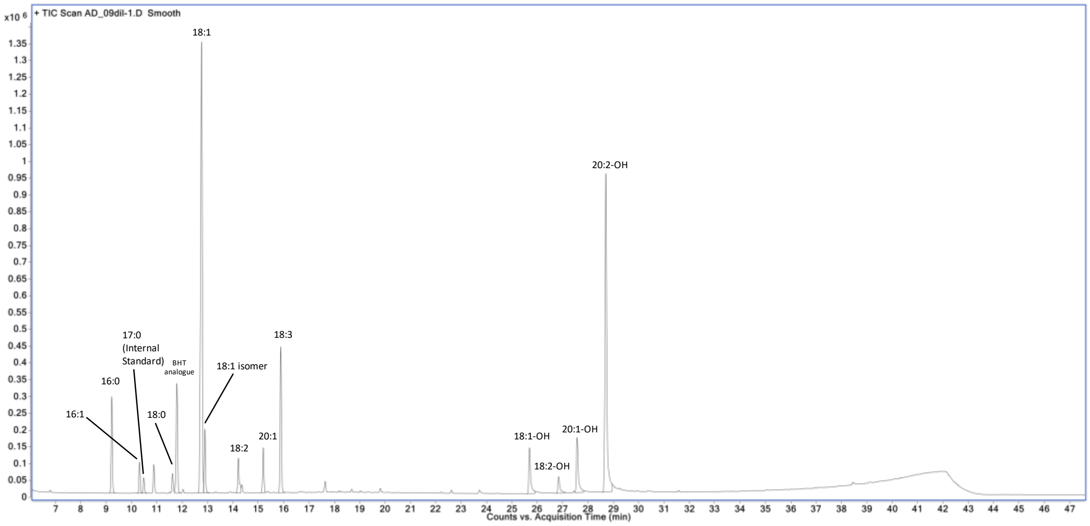

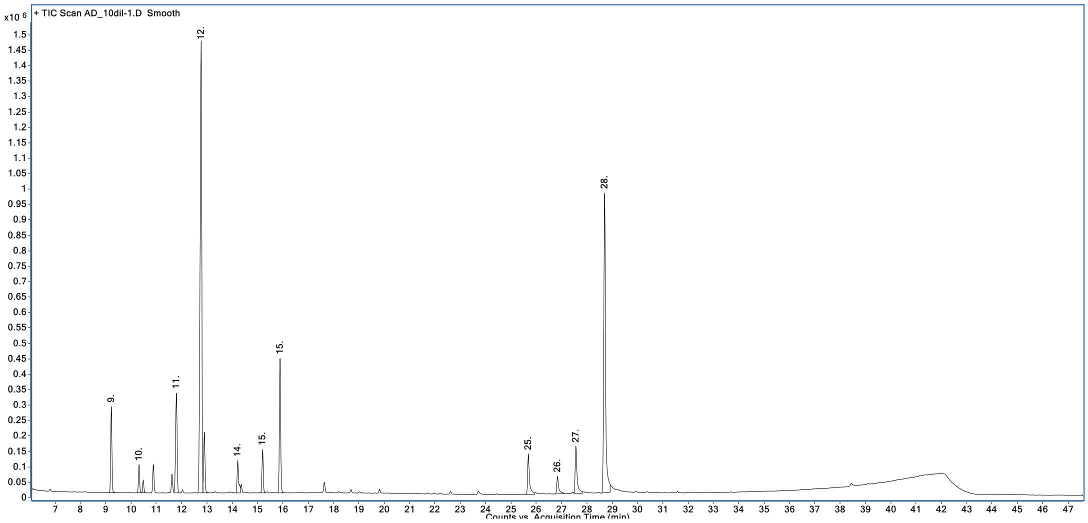

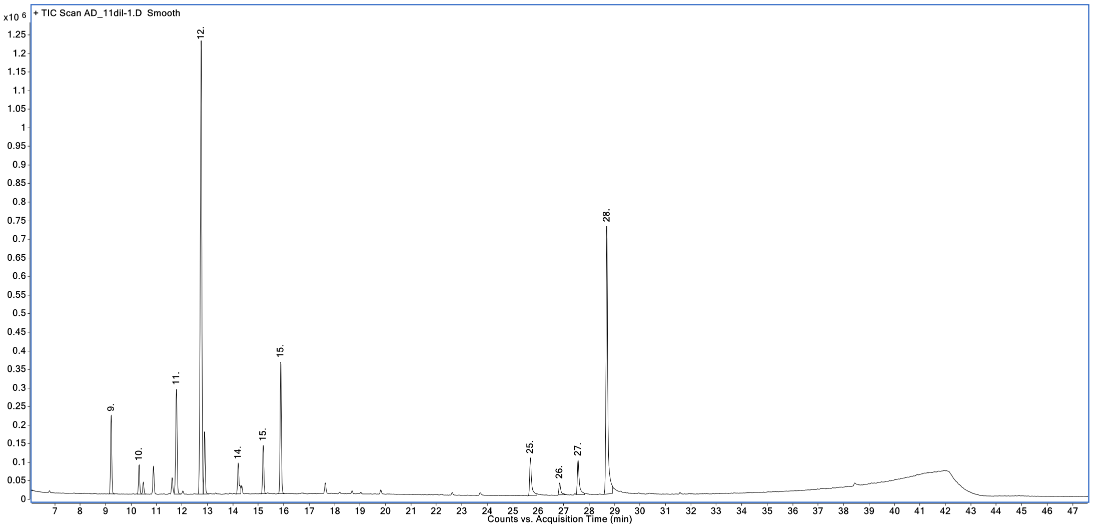

2.5 Mass spectra of 18:1-OH (ricinoleic acid methyl ester)

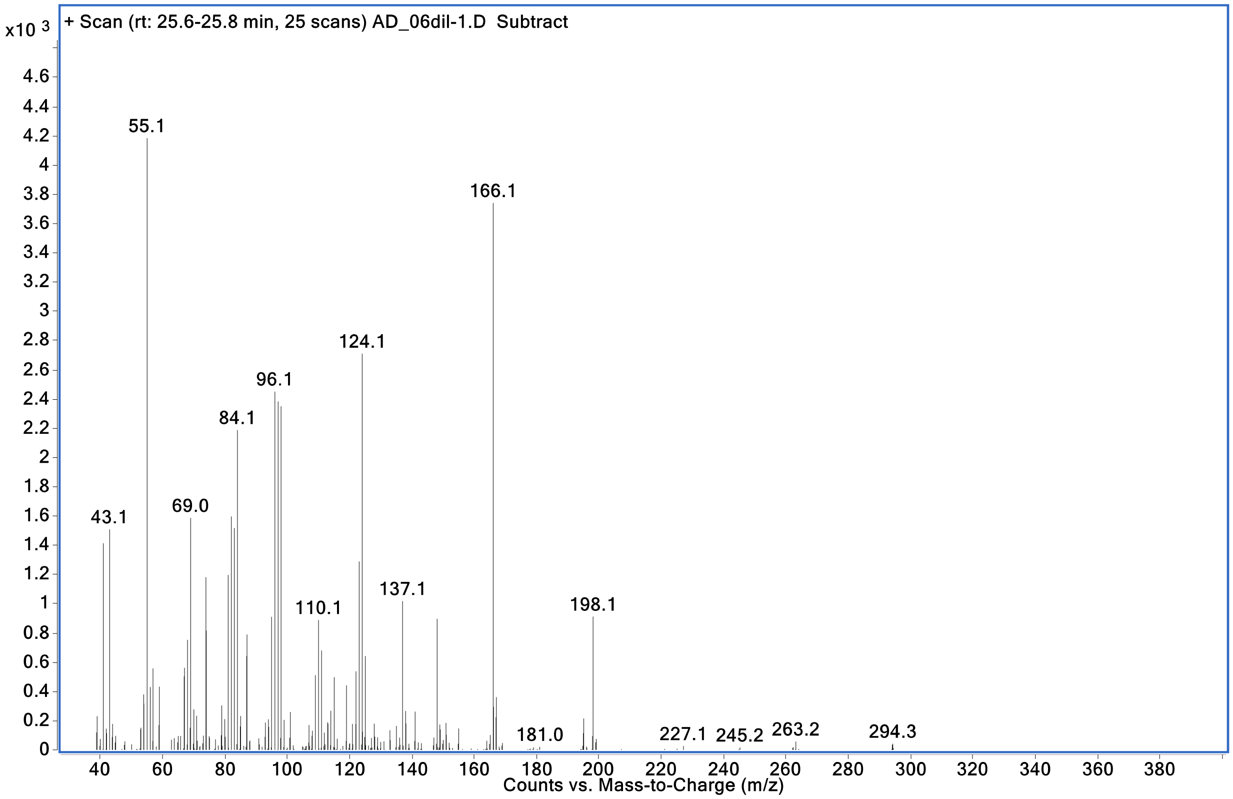

2.6 Mass spectra of 18:2-OH (densipolic acid methyl ester)

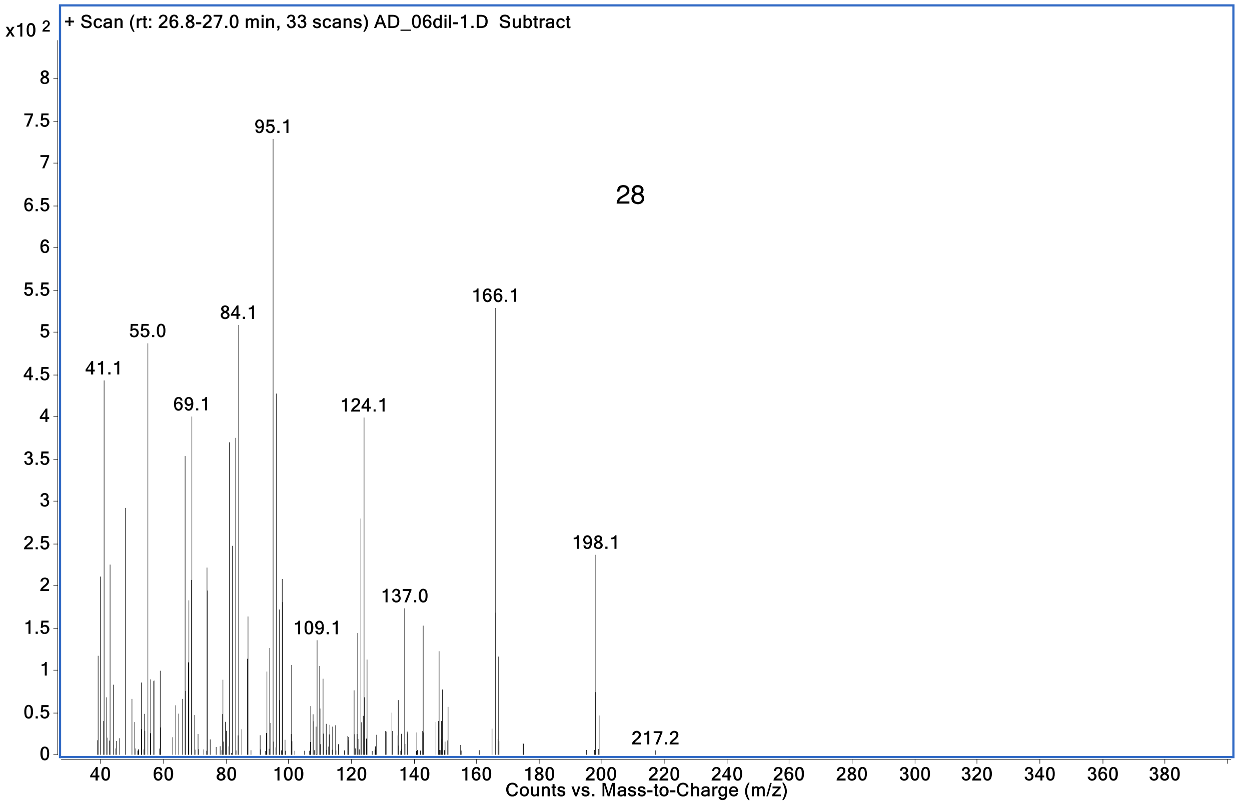

2.7 Mass spectra of 20:1-OH (lesquerolic acid methyl ester)

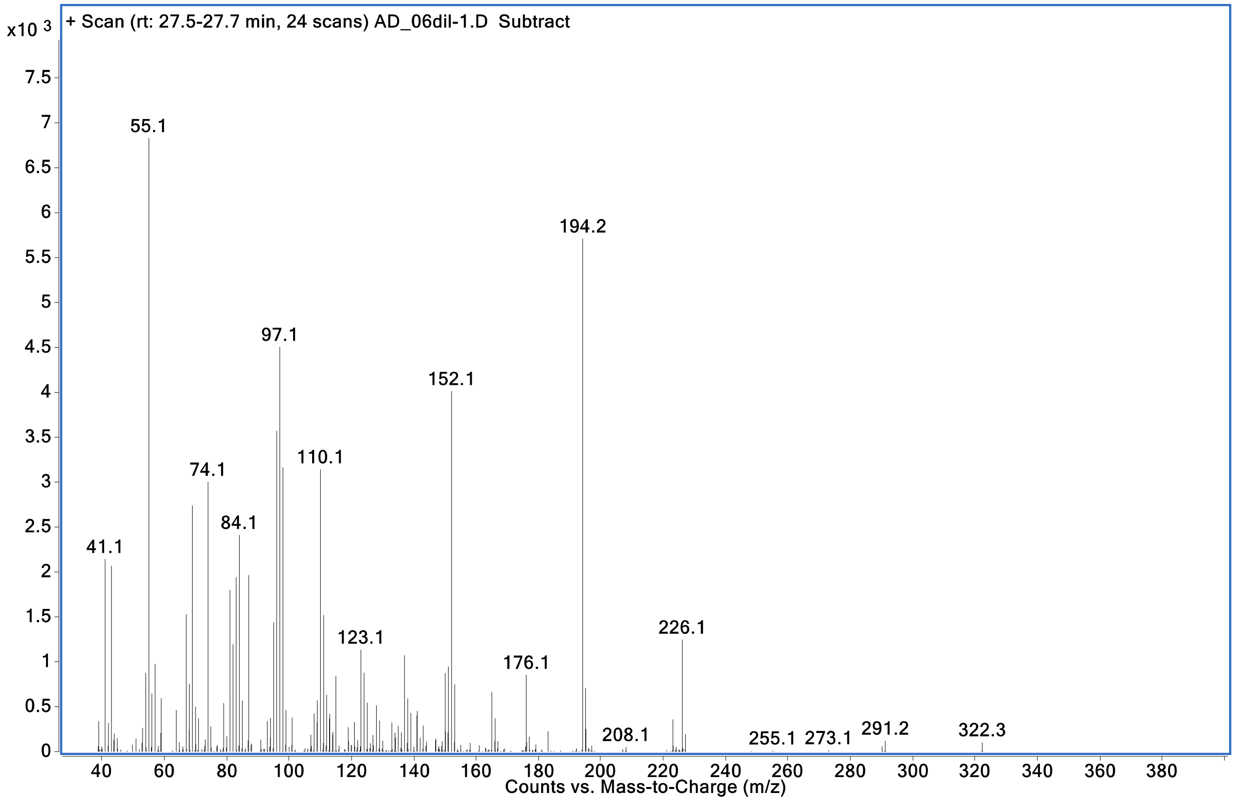

2.8 Mass spectra of 20:2-OH (auricolic acid methyl ester)

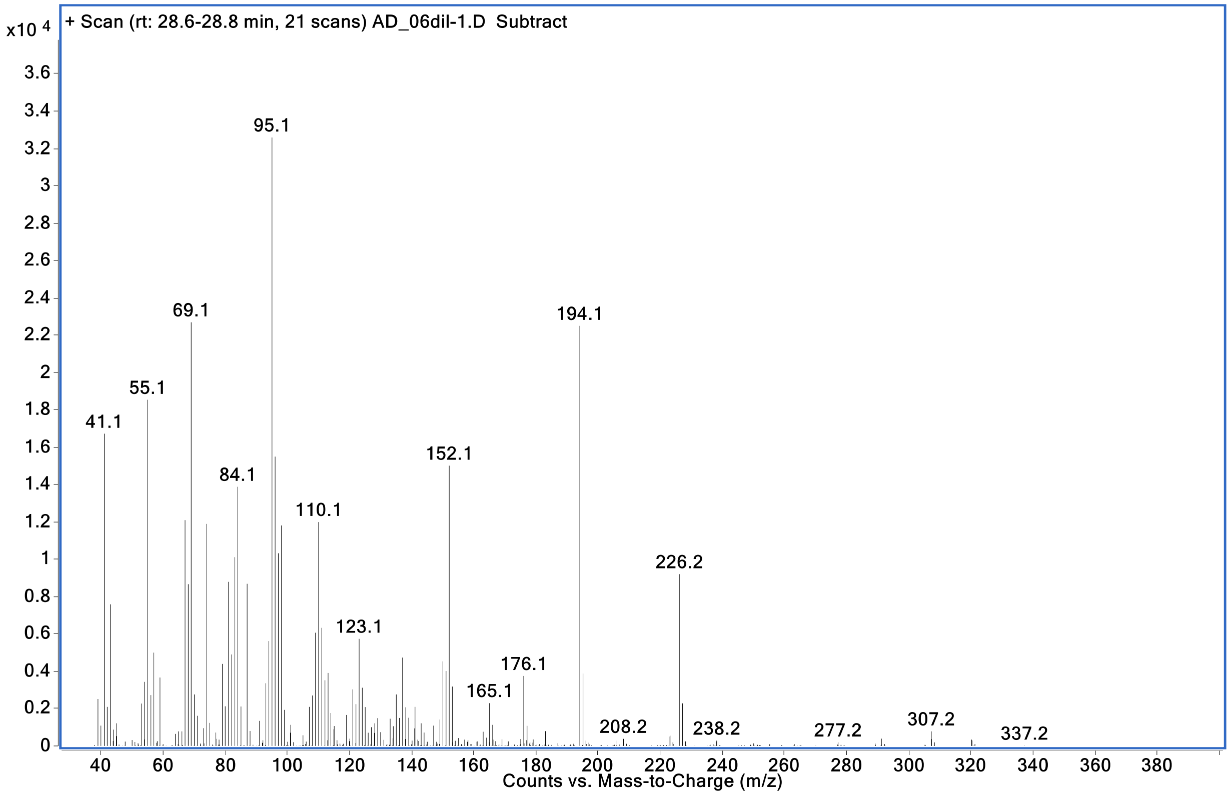

2.9 Mass spectra of 16:0 (palmitic acid)

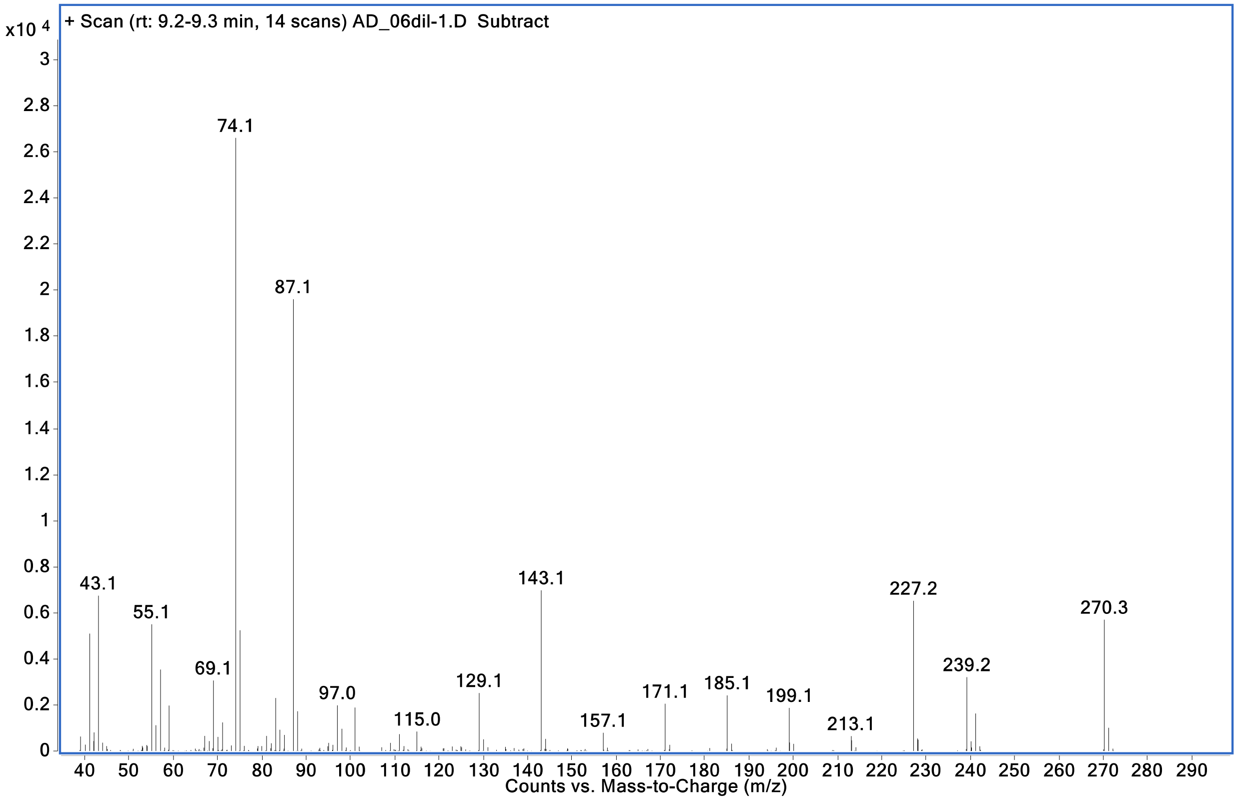

2.10 Mass spectra of 16:1 (palmitoleic acid)

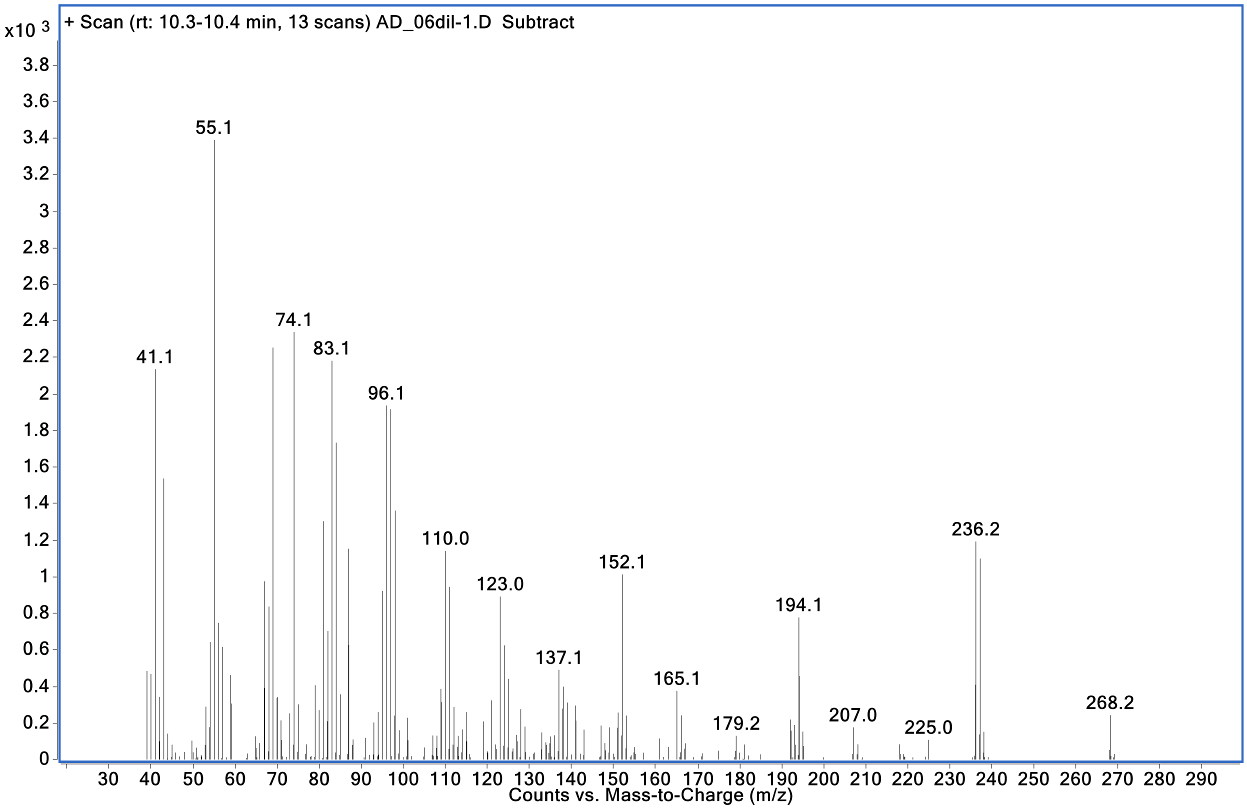

2.11 Mass spectra of 18:0 (oleic acid)

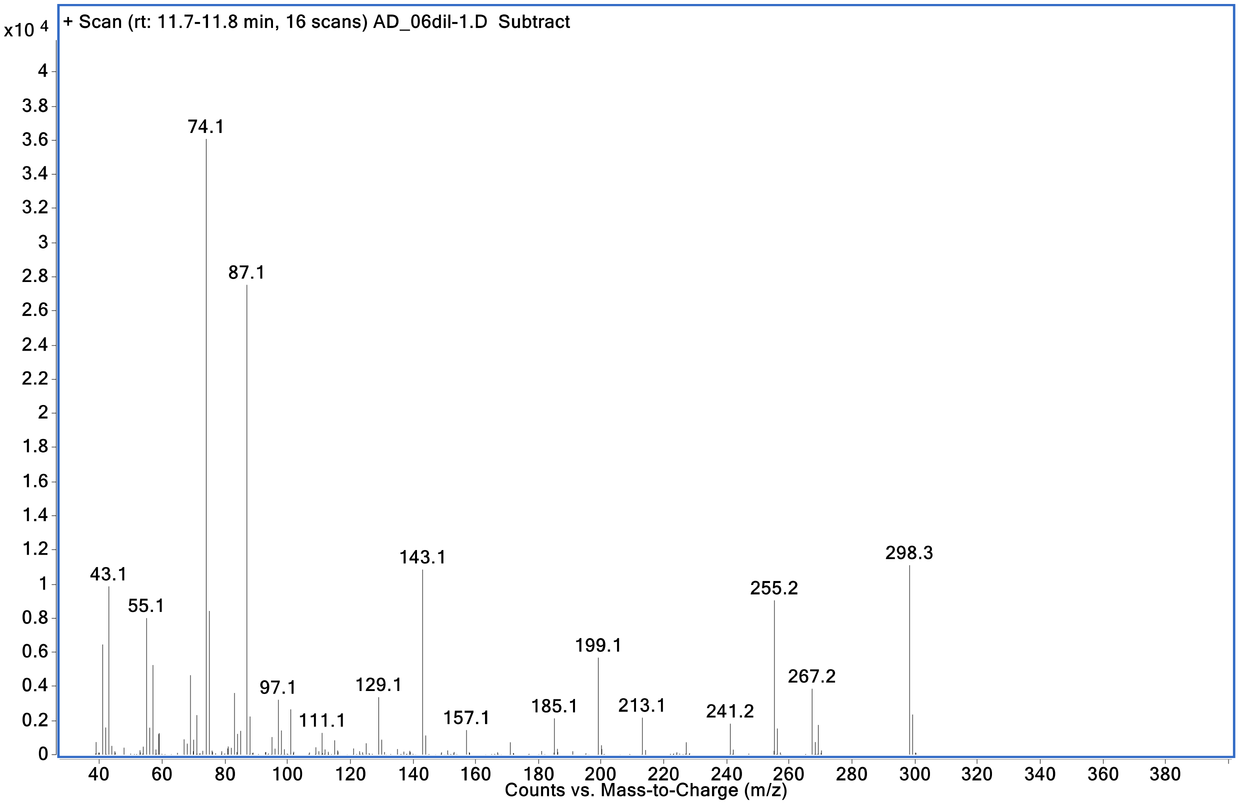

2.12 Mass spectra of 18:1 (stearic acid)

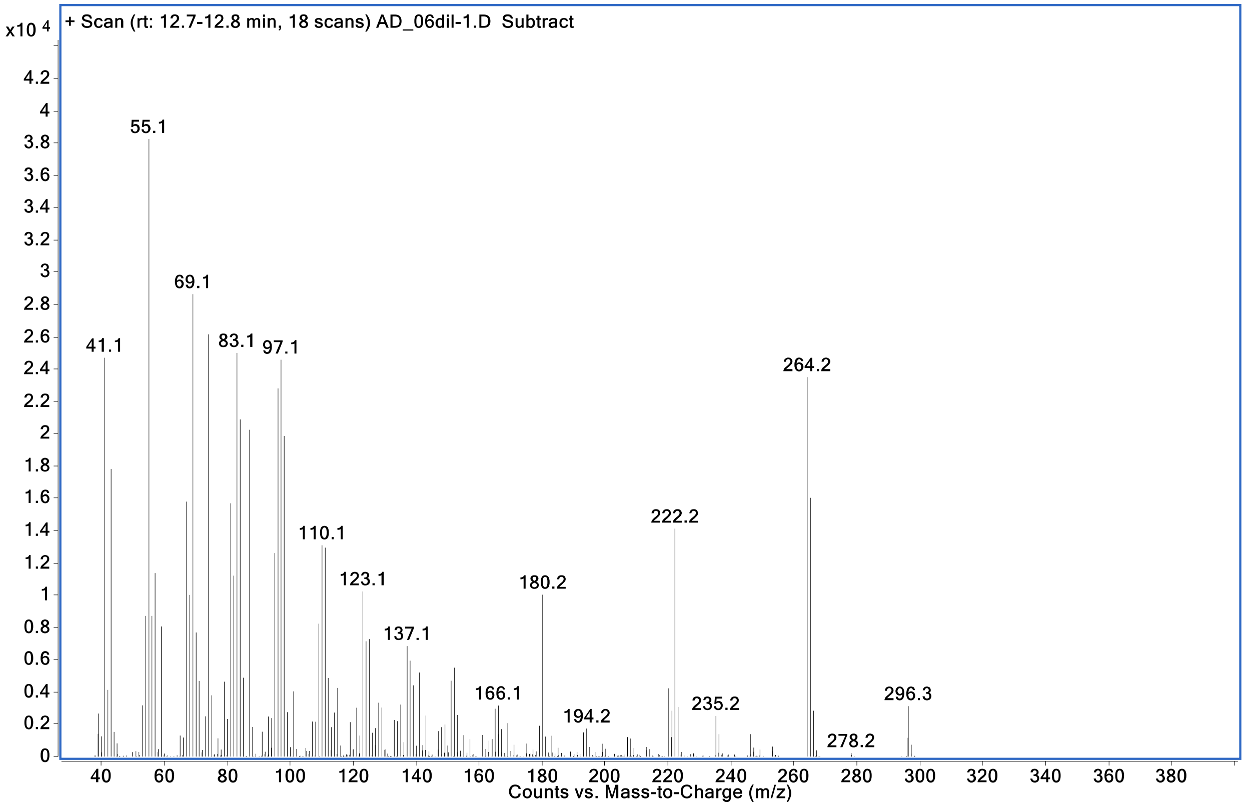

2.13 Mass spectra of 18:1 isomer (stearic acid isomer)

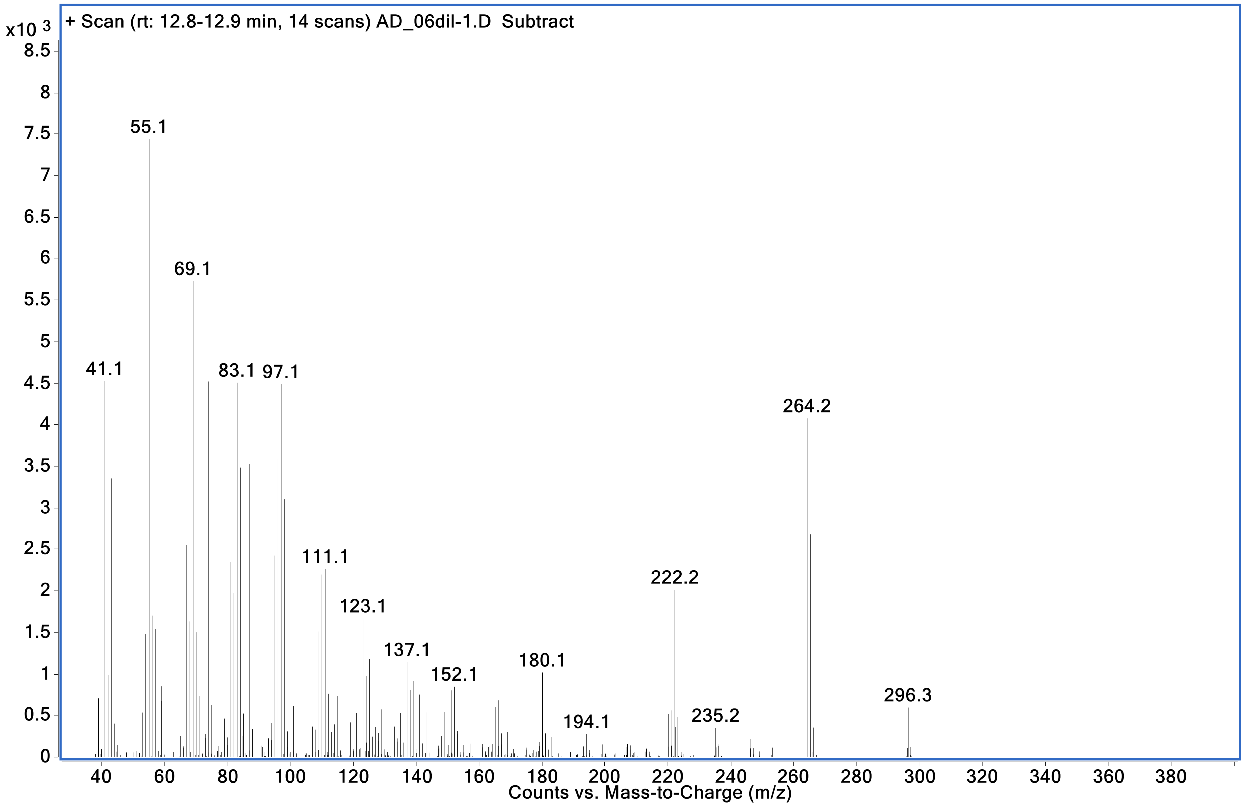

2.14 Mass spectra of 18:2 (linoleic acid)

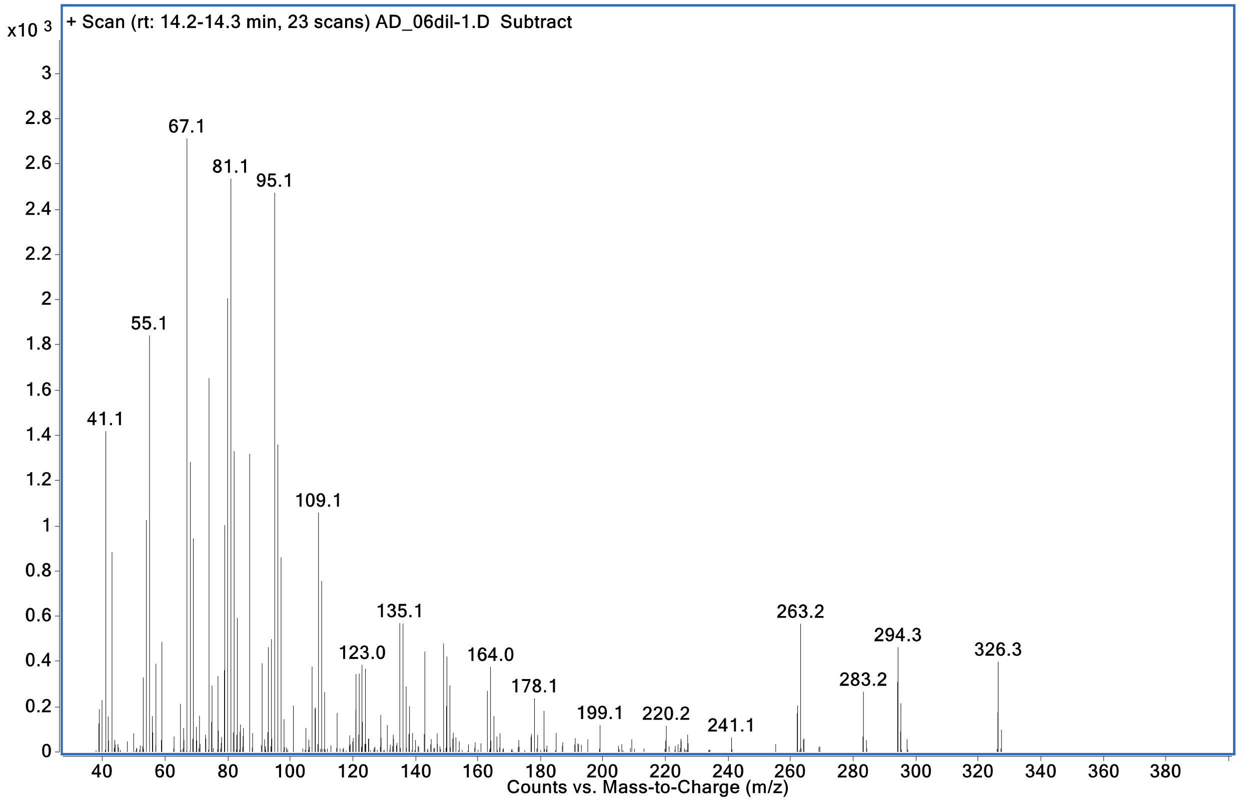

2.15 Mass spectra of 18:3 (linolenic acid)

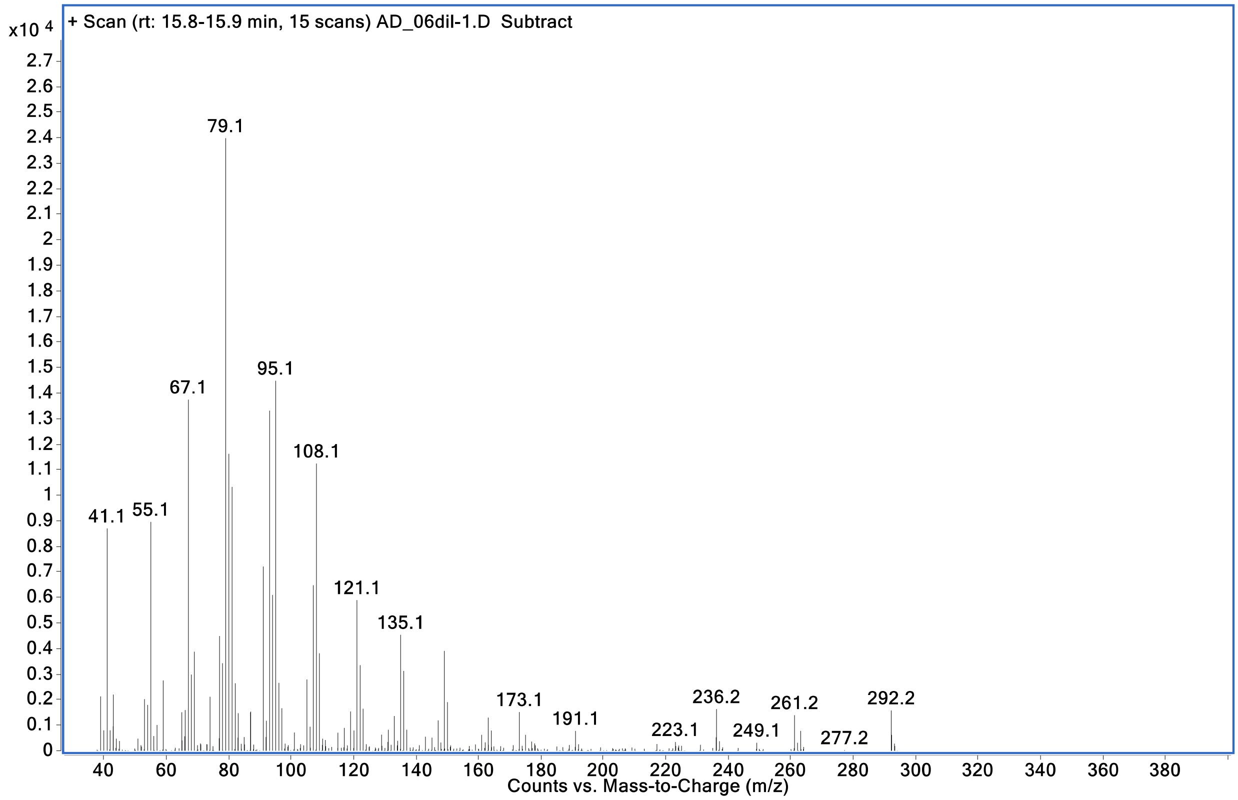

2.16 Mass spectra of 20:1 (eicosenoic acid)

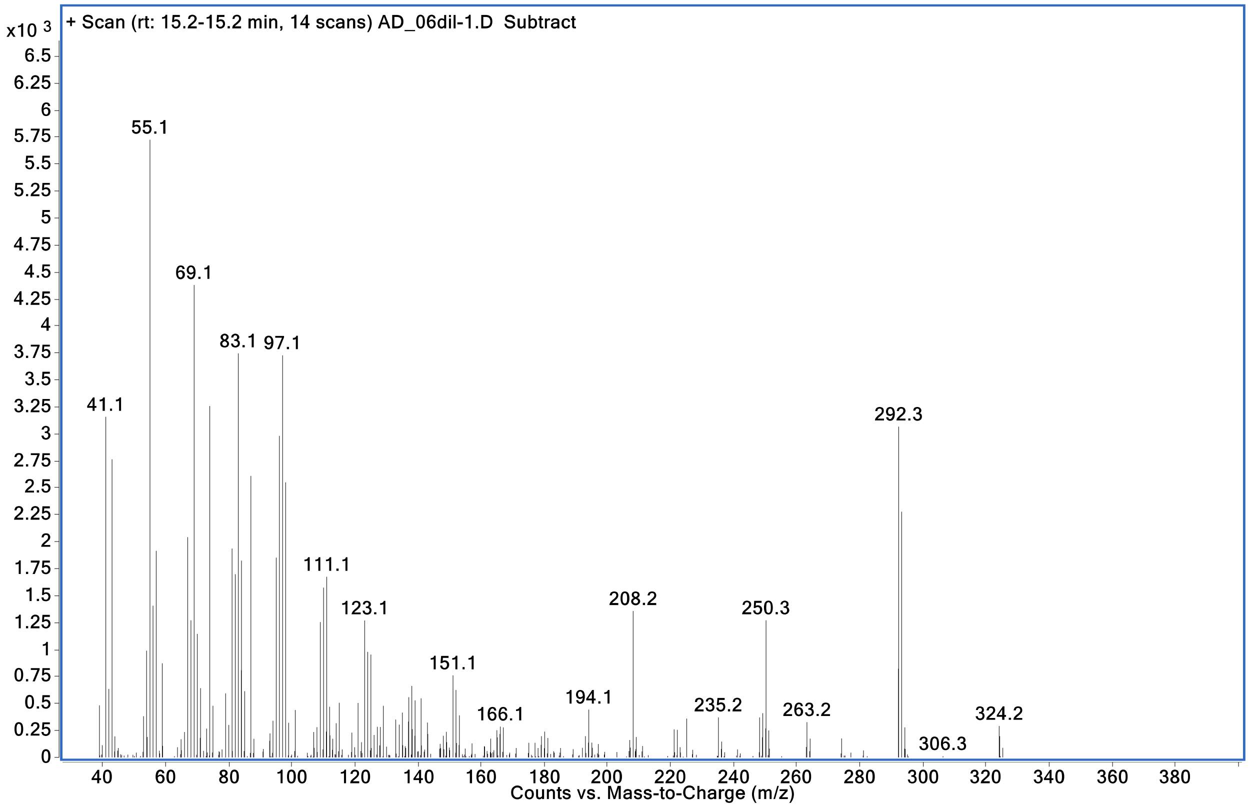

Supplement: Supplementary file 1 [file DataSheet_1.pdf]
