# Supplementary material for: Transcriptomic analysis of seed development in Paysonia auriculata (Brassicaceae) identifies genes involved in hydroxy fatty acid biosynthesis
Source: Front Plant Sci. 2023 Jan 13;13:1079146. doi: 10.3389/fpls.2022.1079146 (PMC9880434; doi:10.3389/fpls.2022.1079146)
Supplement: Supplementary file 2 [file DataSheet_2.docx]

Supplementary Material

# Supplementary Figures and Tables

## Supplementary Figures

**Supplementary Figure 1.** Seed area of individual siliques collected at different times over development. Siliques were collected from weeks 2 to 8 after pollination. Each mean and confidence interval represent a single silique. Error bars represent 95% confidence intervals.


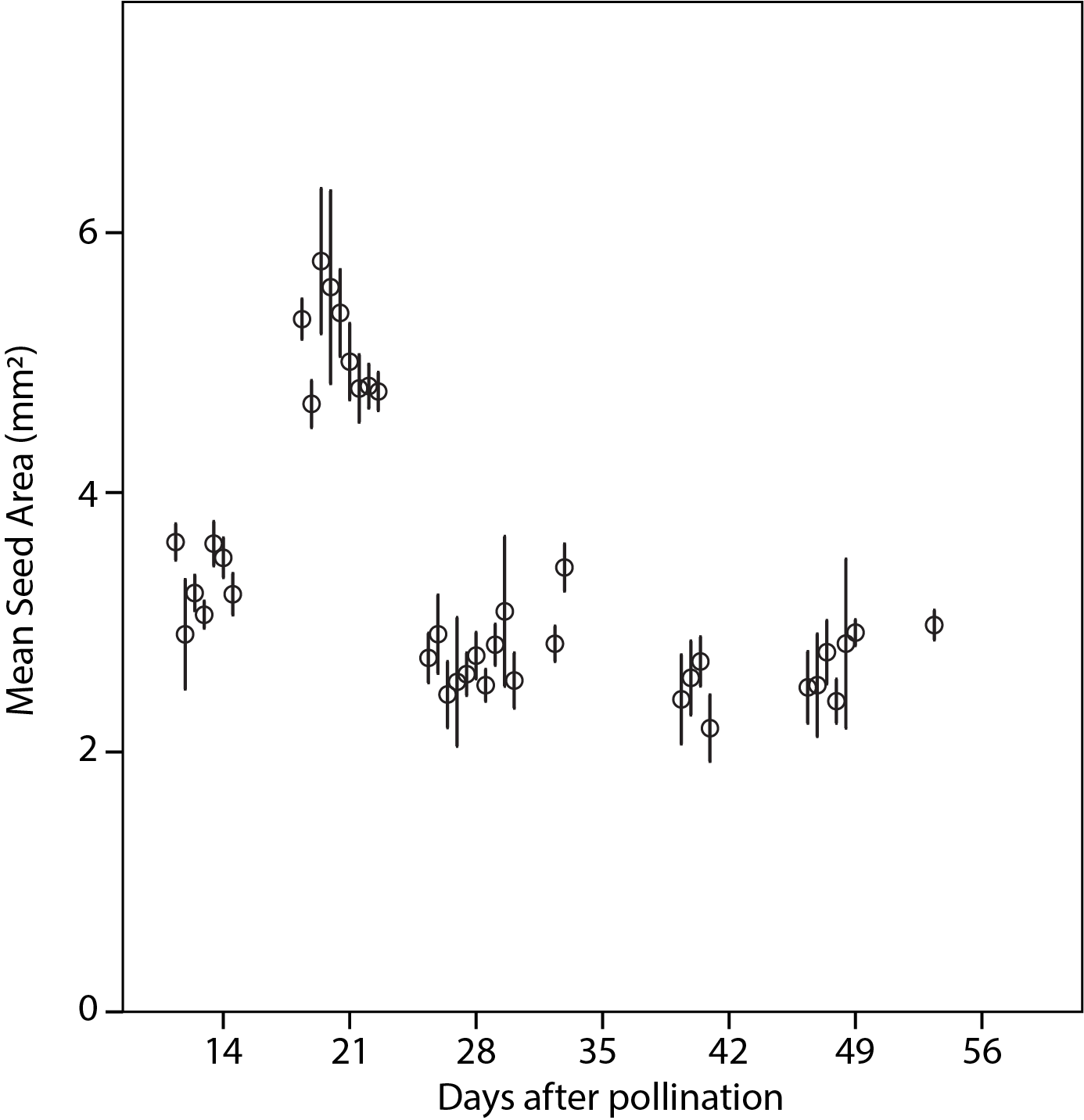


**Supplementary Figure 2.** Seed number of individual siliques collected at different times over development. Filled in circles are actual numbers in individual siliques, open diamond is mean, bars and whiskers are 95% confidence intervals.


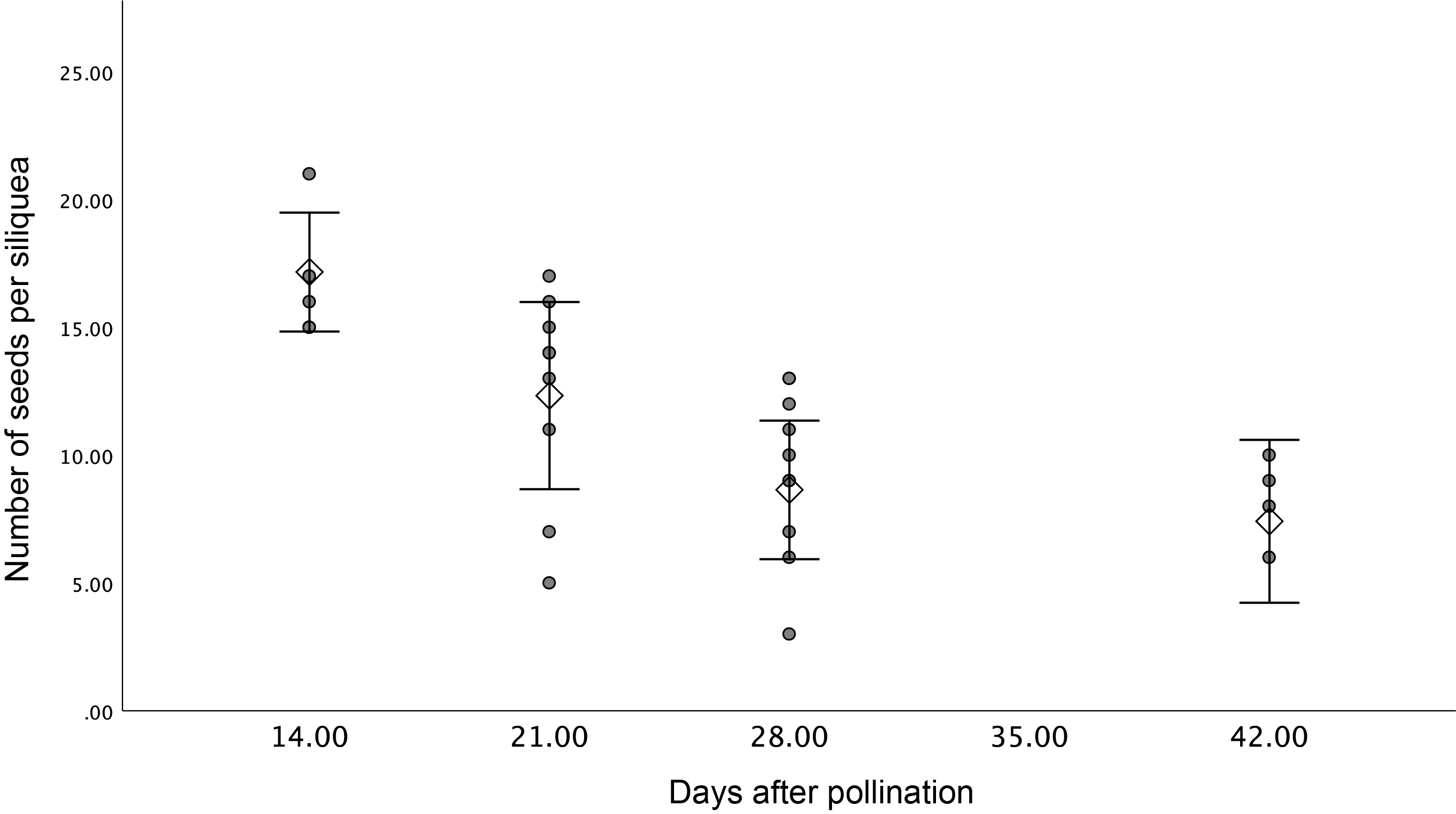


**Supplementary Figure 3.** Phylogenetic tree of FAH12 in various species.

PfFAH12 (AAC32755), PlFAH12 (ABQ01458), OvFAD2-2 (APQ40636), HbFAH12-1 (AGN95845), HbFAH12-2 (AGN95846), RcFAH12 (XP_002528127), CpFAH12 (BAW27658),


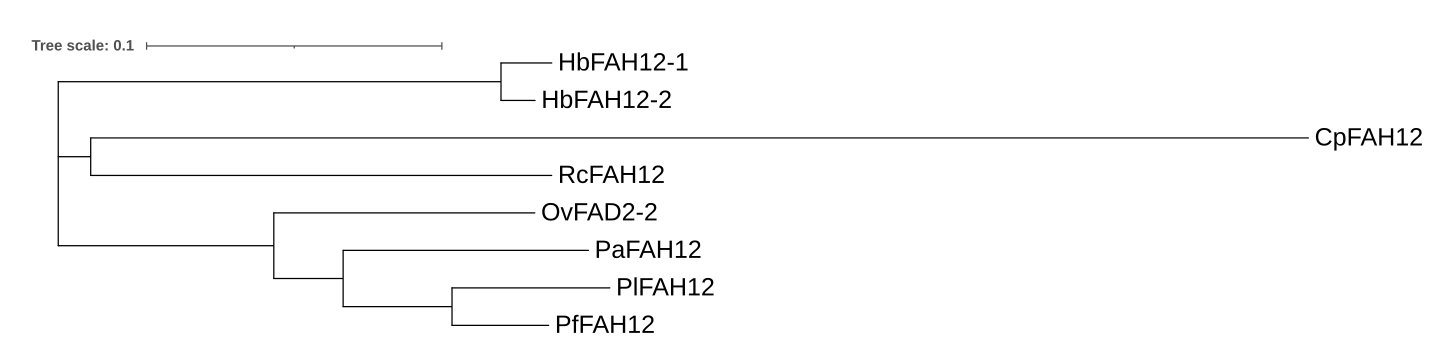


**Supplementary Figure 4.** Phylogenetic tree of FAD2 in various species.

PfFAD2 (AFP21683), AtFAD2 (NP_001319529), OvFAD2-1 (APQ40635), HbFAD2-1 (AGN95841), HbFAD2-2 (AGN95842), HbFAD2-3 (AGN95843), RcFAD2 (NP_001310648), CpFAD2 (ABS18716).

**
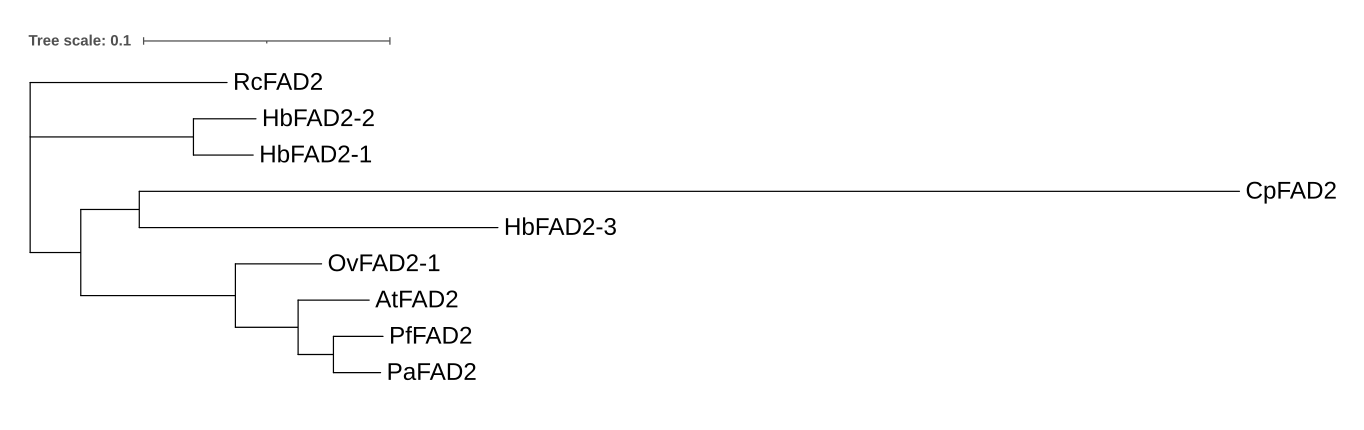
**

**Supplementary Figure 5.** Phylogenetic tree of FAD3 in various species.

PfFAD3-1 (AWX65624), PfFAD3-2 (AWX65625), PlFAD3-1 (UIH10881), PlFAD3-2 (UIH10882), AtFAD3 (AT2G29980), CsFAD3 (ABA55806), OvFAD3 (AWY93817), RcFAD3 (EEF36775).

**
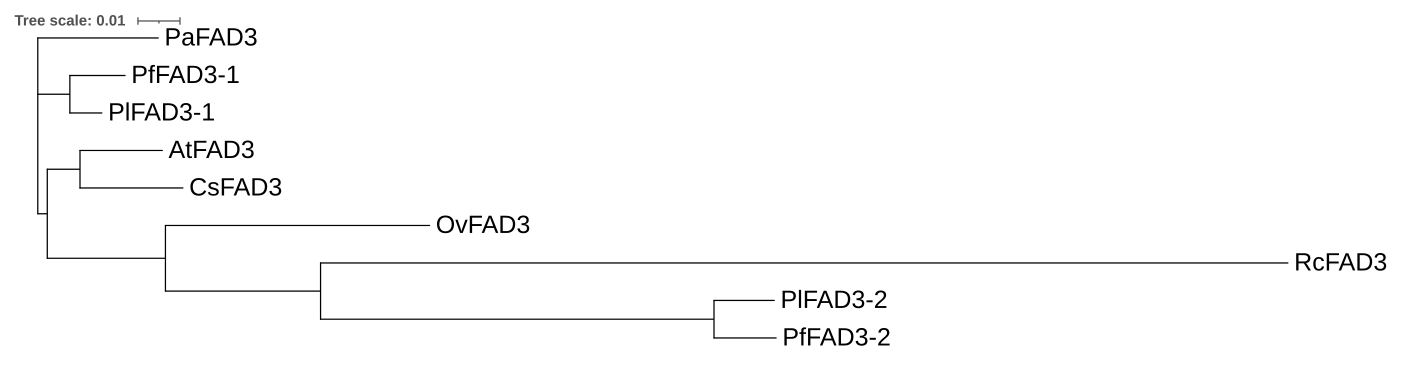
**

**Supplementary Figure 6.** Amino acid alignment of FAD3 in various species.

PfFAD3-1 (AWX65624), PfFAD3-2 (AWX65625), PlFAD3-1 (UIH10881), PlFAD3-2 (UIH10882), AtFAD3 (AT2G29980), CsFAD3 (ABA55806), OvFAD3 (AWY93817), RcFAD3 (EEF36775). His-boxes were highlighted in yellow.

**Supplementary Figure 7.** Phylogenetic tree of KCS18 in various species.

CsKCS18-A (ADN10814), CsKCS18-B (ADN10812), CsKCS18-C (ADN10813), AtKCS18 (AT4G34520), PfKCS18 (AAK62348), RcKSC18 (XP_002516167), OvKCS18 (APQ40637).


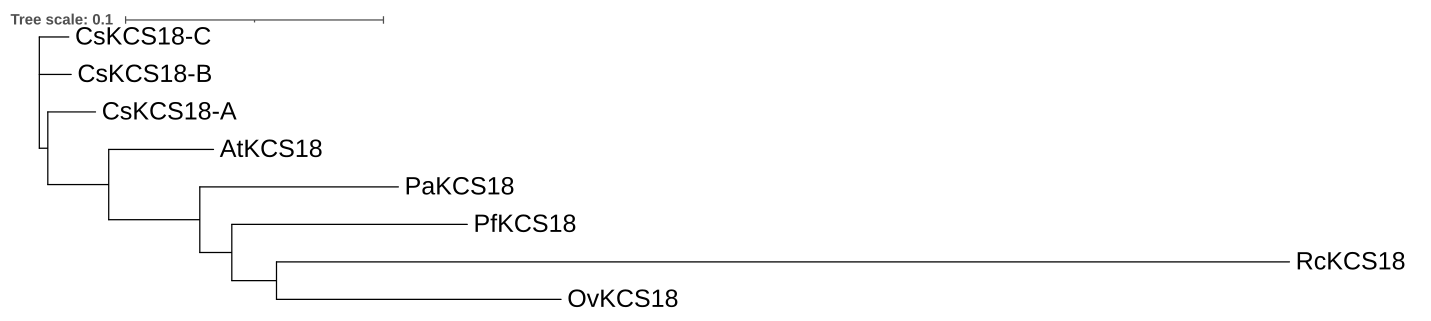


**Supplementary Figure 8.** Amino acid alignment of KCS18 in various species.

CsKCS18-A (ADN10814), CsKCS18-B (ADN10812), CsKCS18-C (ADN10813), AtKCS18 (AT4G34520), PfKCS18 (AAK62348), RcKSC18 (XP_002516167), OvKCS18 (APQ40637).

The colored regions indicated conserved amino acids or sequences, a Gly-Gly region containing a putative active Cys site was highlighted in blue.

**Supplementary Figure 9.** Phylogeny of FAD2 and FAH12 genes in the family Brassicaceae.

FAH12 sequences are in bold, and the branch leading to the FAH12 clade is in red. Length of the tree is indicated by substitution rate at base. Support values are from 1000 bootstrapped analyses. Sequences used in this analysis: FAD2: *A. helleri* (Araha.1578s0006.1), *A. thaliana* (NM_112047.4), *B. rapa* (Brara.E02874.1), *B. stricta* (Bostr.19424s1149.1), *C. glabrata* (KJ573490.1), *C. grandiflora* (Cagra.0430s0042.1), *C. rubella* (Carubv10013933m), *C. sativa* (GU929417.1), *C. sativus* (Cucsa.242720.2), *C. sinensis* (orange1.1g016781m), *E. salsugineum* (Thhalv10020914m), *F. vesca* (mrna22642.1-v1.0-hybrid), *L. campestre* (FJ907546.1), *P. auriculata* (MT496776), *P. fendleri* (KC972621.1), *S. alba* (KY305535.1), *T. hassleriana* (XM_010559645.1). FAH12: *P. auriculata* (MT496774), *P. fendleri* (KC972619.1), *P. lindheimeri* (EF432246.1).


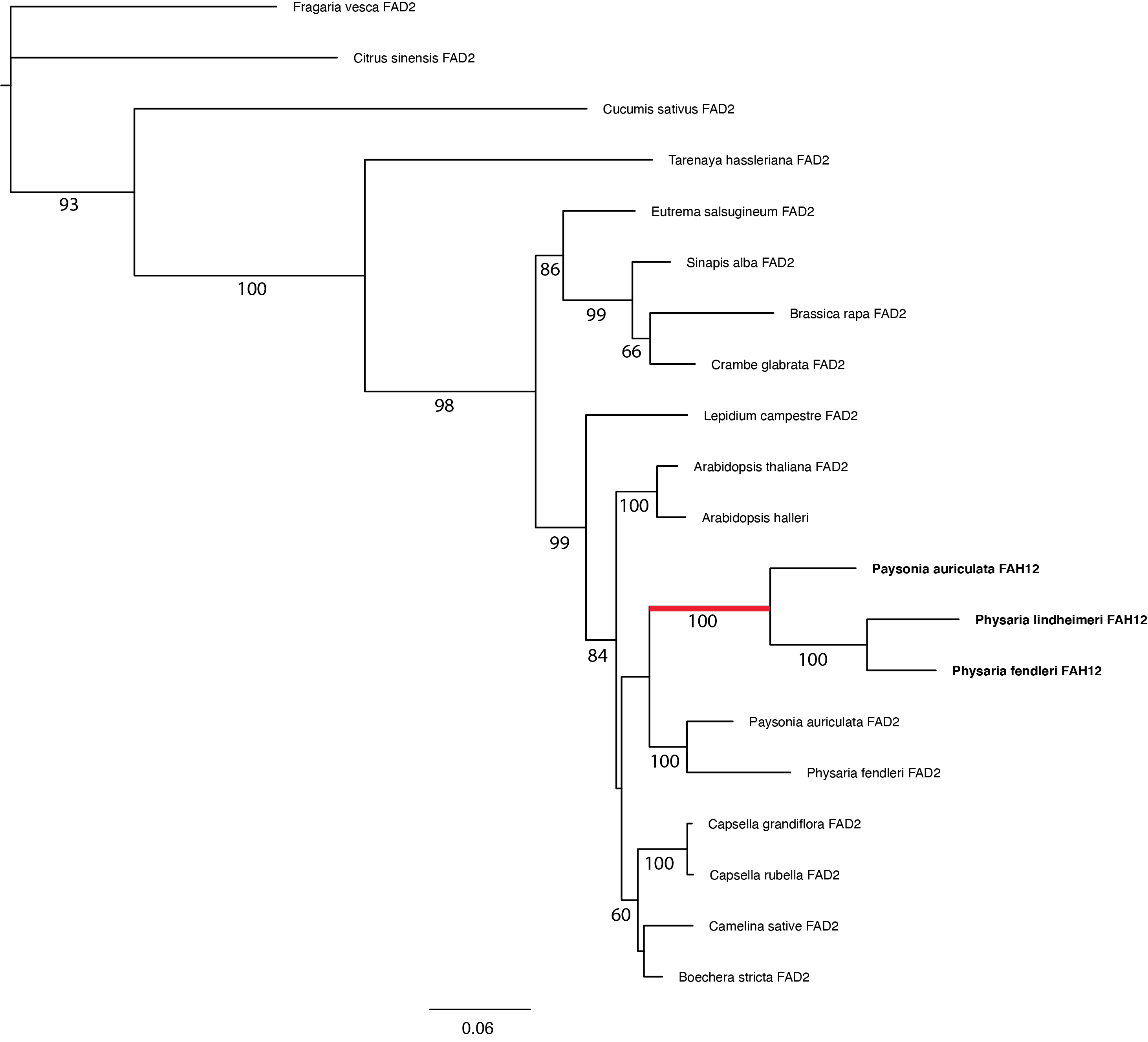


## Supplementary Tables

**Supplementary Table 1. Summary statistics of sequencing data of individual library**

| Sample | Number of raw reads | Number of filtered reads | Overall alignment rate to Trinity assembly using Bowtie2 |
| --- | --- | --- | --- |
| Pa_2_rep1 | 39,905,282 | 21,876,307 | 82.38% |
| Pa_2_rep2 | 31,471,350 | 16,989,546 | 82.43% |
| Pa_2_rep3 | 38,260,148 | 22,229,570 | 81.02% |
| Pa_3_rep1 | 37,529,310 | 22,195,302 | 78.40% |
| Pa_3_rep2 | 41,054,622 | 23,754,920 | 79.20% |
| Pa_3_rep3 | 39,799,045 | 21,283,284 | 77.88% |
| Pa_4_rep1 | 38,615,229 | 20,826,262 | 78.78% |
| Pa_4_rep2 | 37,044,630 | 20,595,350 | 80.17% |
| Pa_4_rep3 | 33,715,960 | 17,721,020 | 79.40% |
| Pa_6_rep1 | 34,663,919 | 19,231,331 | 78.35% |
| Pa_6_rep2 | 39,465,750 | 19,866,715 | 83.25% |
| Pa_6_rep3 | 32,607,593 | 18,749,052 | 78.08% |

**Supplementary Table 2. Summary statistics of *de novo* transcriptome assembly with Trinity**

| Trinity *de novo* assembly |  |
| --- | --- |
| Total assembled bases | 226,807,152 |
| Number of transcripts | 270,114 |
| Number of isoforms | 122,730 |
| Average transcript length | 840 bp |
| Max gene length | 18,345 bp |
| Min gene length | 201 bp |
| Transcript N50 | 1,255 bp |
| GC content | 40.80 |
| Length and number distribution of isoforms | |
| 200 – 400 | 67,710 |
| 400 – 600 | 17,198 |
| 600 – 800 | 9,376 |
| 800 – 1,000 | 6,408 |
| 1,000 – 1,500 | 10,313 |
| > 1500 | 11,725 |
| Total | 122,730 |

**Supplementary Table 3. GO enrichments of DE genes from different clusters in SOM.**

| **GO** | **Term** | **Cluster 1** | **Cluster 2** | **Cluster 3** | **Cluster 4** |
| --- | --- | --- | --- | --- | --- |
| GO:0009414 | response to water deprivation | 6.4 | 6.5 | 1.5 | 6.9 |
| GO:0009416 | response to light stimulus | 4.3 | 6.0 | 2.4 | 0.4 |
| GO:0055114 | oxidation-reduction process | 14.4 | 19.1 | 8.9 | 6.8 |
| GO:1901700 | response to oxygen-containing compound | 5.2 | 9.4 | 9.2 | 4.1 |
| GO:0000302 | response to reactive oxygen species | 0.5 | 7.7 | 4.1 | 0.3 |
| GO:0006979 | response to oxidative stress | 3.3 | 11.6 | 6.3 | 1.3 |
| GO:0042446 | hormone biosynthetic process | 0.3 | 5.3 | 0.3 | 0.2 |
| GO:0030258 | lipid modification | 0.0 | 14.5 | 0.0 | 0.3 |
| GO:0044242 | cellular lipid catabolic process | 0.0 | 23.8 | 0.3 | 0.1 |
| GO:0006996 | organelle organization | 0.0 | 3.2 | 0.0 | 0.0 |
| GO:0019222 | regulation of metabolic process | 0.0 | 11.2 | 0.0 | 0.0 |
| GO:0080090 | regulation of primary metabolic process | 0.0 | 8.0 | 0.0 | 0.0 |
| GO:0046907 | intracellular transport | 0.0 | 3.8 | 0.0 | 0.0 |
| GO:0015031 | protein transport | 0.0 | 5.0 | 0.0 | 0.0 |
| GO:0007031 | peroxisome organization | 0.0 | 12.2 | 0.0 | 0.0 |
| GO:0007568 | aging | 0.0 | 1.1 | 2.0 | 5.1 |
| GO:0005985 | sucrose metabolic process | 1.0 | 0.1 | 2.2 | 9.3 |
| GO:0055085 | transmembrane transport | 1.6 | 0.4 | 5.9 | 4.1 |
| GO:0010876 | lipid localization | 1.6 | 0.0 | 5.4 | 5.0 |
| GO:0019915 | lipid storage | 0.4 | 0.1 | 5.8 | 2.0 |
| GO:0090351 | seedling development | 0.1 | 0.5 | 7.7 | 2.2 |
| GO:0009737 | response to abscisic acid | 2.3 | 2.2 | 3.6 | 2.0 |
| GO:1901568 | fatty acid derivative metabolic process | 0.2 | 0.2 | 4.9 | 0.0 |
| GO:0010345 | suberin biosynthetic process | 0.0 | 0.1 | 9.1 | 0.0 |
| GO:0033993 | response to lipid | 2.0 | 1.2 | 3.2 | 1.2 |
| GO:0009698 | phenylpropanoid metabolic process | 5.0 | 0.0 | 21.1 | 0.6 |
| GO:0009725 | response to hormone | 6.8 | 1.0 | 7.4 | 1.5 |
| GO:0006629 | lipid metabolic process | 9.7 | 4.8 | 9.0 | 3.3 |
| GO:0008202 | steroid metabolic process | 11.0 | 0.3 | 3.4 | 1.5 |
| GO:1901658 | glycosyl compound catabolic process | 7.7 | 0.0 | 2.2 | 0.0 |
| GO:0071555 | cell wall organization | 14.0 | 0.0 | 3.8 | 0.1 |
| GO:0006633 | fatty acid biosynthetic process | 14.9 | 0.0 | 8.2 | 1.8 |
| GO:0009813 | flavonoid biosynthetic process | 9.3 | 0.2 | 4.4 | 1.3 |
| GO:0008610 | lipid biosynthetic process | 14.6 | 0.2 | 6.3 | 2.8 |
| GO:0006637 | acyl-CoA metabolic process | 5.0 | 0.0 | 1.1 | 1.0 |
| GO:0044264 | cellular polysaccharide metabolic process | 12.6 | 0.0 | 1.2 | 0.9 |
| GO:0006733 | oxidoreduction coenzyme metabolic process | 13.5 | 0.6 | 0.0 | 0.0 |
| GO:0016051 | carbohydrate biosynthetic process | 17.5 | 0.1 | 0.4 | 0.5 |
| GO:0016049 | cell growth | 2.5 | 0.0 | 0.1 | 0.0 |
| GO:0010191 | mucilage metabolic process | 12.1 | 0.0 | 0.1 | 0.0 |
| GO:0010154 | fruit development | 4.2 | 1.5 | 1.7 | 1.4 |
| GO:0016053 | organic acid biosynthetic process | 14.6 | 0.9 | 2.9 | 0.2 |
| GO:0010214 | seed coat development | 9.5 | 0.1 | 1.0 | 0.0 |
| GO:0005976 | polysaccharide metabolic process | 14.9 | 0.0 | 1.8 | 0.3 |
| GO:0019684 | photosynthesis, light reaction | 22.4 | 0.0 | 1.9 | 0.3 |

White to red color keys indicate negative log10-transformed *p*-values for enrichment (*p* < 0.05).

**Supplementary Table 4. Primers used in cloning and qRT-PCR**

| Gene | Sequence (5’ – 3’) | Purpose |
| --- | --- | --- |
| PaFAH12 | F: CACCATGGGTGCTGGTGGAAGAAT  R: TCATAACTTATTGTTGAACCAGTAG | Cloning |
| PaFAD2 | F: CACCATGGGTGCTGGTGGAAGAAT  R: TCATAACTTATTGTTGTACCAGTACA | Cloning |
| PaFAD3 | F: CACCATGGTGGTTGCTATGG  R: TTAATTGATTTTAGATTTGTC | Cloning |
| PaKCS18 | F: CACCATGACGTCCGTGAACGTA  R: TCAAGACCGCCCATTTTGG | Cloning |
| PaFAH12 | F: ATTACTTGGCTTGGCCCCTC  R: CGTGACCAATGACCCAGACA | qRT-PCR |
| PaFAD2 | F: CACGCTTTTAGCAACTACCAGT  R: TCGAGGGATCCTGTGTTGGA | qRT-PCR |
| PaFAD3 | F: GCGCTGGAGATCCGAAGAAA  R: GATCTTAAACGGCGGCTGTG | qRT-PCR |
| PaKCS18 | F: ATCAACAATCGATGTCGAAGCA  R: TCCCAATGCAATCTGCCAAATC | qRT-PCR |
| 18S | F: GAGAAACGGCTACCACATCCA  R: CCGTGTCAGGATTGGGTAATTT | qRT-PCR |
